# Transgender identity and psychiatric care in Italian prisons: a thematic analysis of systemic gaps and institutional challenges

**DOI:** 10.3389/fpsyt.2026.1737418

**Published:** 2026-02-02

**Authors:** Paolo Meneguzzo, Daniele Zuccaretti, Matilde Obici, Marco Cristoforetti, Alberto Scala, Marina Bonato, Marina Miscioscia, Angela Favaro, Andrea Garolla

**Affiliations:** 1 Department of Neuroscience, University of Padova, Padova, Italy; 2 Padova Neuroscience Center, University of Padova, Padova, Italy; 3 Regional Reference Center for Gender Incongruence, Azienda Ospedale-Università di Padova, Padova, Italy; 4 AULSS 1 Dolomiti, Belluno, Italy; 5 Hygiene and Public Health Unit, Department of Cardiac Thoracic Vascular Sciences and Public Health, University of Padova, Padova, Italy; 6 Unit of Andrology and Reproductive Medicine, Department of Medicine, University of Padova, Padova, Italy; 7 Department of Developmental Psychology and Socialization, University of Padova, Padova, Italy

**Keywords:** gender affirming hormonal therapy (GAHT), gender diverse, minority stress, prison, protective house, psychiatric care, transgender

## Abstract

**Background:**

Transgender and gender-diverse (TGD) individuals face high levels of stigma, discrimination, and psychological distress, which are amplified in carceral settings. Italian prisons have introduced specialized sections for TGD inmates; however, limited empirical evidence exists on their function and the psychiatric, social, and institutional challenges emerging within them.

**Methods:**

A case-informed qualitative design was applied to four TGD inmates housed in a specialized prison section in Northern Italy. Data were derived from routine psychiatric and endocrinological assessments, administrative documentation, informal and semi-structured interviews, and multidisciplinary team observations. A thematic cross-case synthesis was conducted to identify institutional determinants of distress and barriers to gender-affirming care.

**Results:**

Three themes emerged. (1) Context-dependent fluidity in gender identity recognition and categorization: institutional frameworks relied on static binary models, contributing to confusion and psychological strain. (2) Intra-group tensions within designated housing: discrepancies in transition pathways, administrative criteria, perceived authenticity, and the presence of sexual minority inmates generated conflict and social exclusion. (3) Gaps in gender-affirming and psychiatric care: logistical barriers, limited staff training, reliance on telemedicine, and inconsistent access to hormone therapy disrupted continuity of care and exacerbated distress.

**Discussion:**

These findings illustrate how rigid systems and ambiguous housing policies may reinforce minority stress and undermine psychiatric well-being. Protective units can inadvertently reproduce exclusion when gender identity and sexual orientation are conflated or when criteria for placement remain unclear. Correctional systems should adopt flexible gender-recognition procedures, implement trauma-informed practices, develop distinct housing policies, establish standardized pathways for gender-affirming care, and provide specialized staff training to ensure dignity, safety, and improved mental health outcomes for TGD inmates.

## 1 Introduction

Gender identity, defined as a person’s internal sense of being male, female, both, neither, or elsewhere along the gender spectrum, plays a central role in psychological well-being (1). Transgender and gender-diverse (TGD) individuals—those whose gender identity does not align with the sex assigned at birth—face significant psychosocial stressors across multiple domains of life (2). These include stigma, discrimination, and systemic exclusion, all of which are known contributors to psychological distress and elevated psychiatric risk (3). When placed within highly regulated and punitive institutions such as prisons, these vulnerabilities are often amplified, resulting in increased psychological distress, heightened exposure to victimization, and institutional neglect among marginalized populations, including TGD individuals (4–6).

Incarceration is recognized as a structurally adverse context, particularly for people who inhabit marginalized identities (4). TGD individuals in prison are at heightened risk of victimization, psychological deterioration, and institutional neglect (7). Despite increasing awareness of these risks, correctional systems across many countries—including Italy—remain largely unprepared to provide gender-affirming care and adequate mental health services (8, 9). Institutional practices often reflect binary and cisnormative assumptions and frequently overlook the complex realities of TGD lives, resulting in systemic misrecognition, disrupted gender-affirmation pathways, and poorly adapted psychiatric care. Importantly, most available research has focused on policy analysis or prevalence data, while fewer studies have examined how these institutional arrangements are experienced and negotiated in everyday clinical and relational contexts.

In Italy, only a limited number of prison facilities—estimated to be fewer than ten nationwide—have established specialized sections for TGD individuals, according to available ministerial reports and policy documents. These sections are intended to provide safer environments and are most often located within male institutions. According to the 2018 Report to Parliament, it was recommended that specific sections for TGD individuals be hosted within female institutions, prioritizing gender identity as a subjective experience rather than merely anatomical characteristics ^1^. However, precise national data on the number and distribution of these sections are not publicly available, and implementation of these recommendations has remained uneven across the country. As a result, most TGD sections continue to operate within male facilities, posing significant risks for inmates whose gender identity does not align with their placement. Moreover, the current system provides limited consideration of the needs of TGD men and non-binary individuals, leaving a portion of the TGD population unprotected. Little empirical work has explored how these organizational choices shape identity recognition, access to psychiatric care, and social dynamics within protective housing units. This study adopts a case-informed, qualitative approach to address this gap by examining the experiences of four TGD inmates housed in specialized prison sections in Italy. Rather than presenting the cases as isolated clinical accounts, we synthesize them to illuminate how institutional rules, interpersonal dynamics, and psychiatric practices intersect in shaping vulnerability, conflict, and care trajectories. Guided by the frameworks of minority stress theory and social psychiatry, this analysis aims to move beyond descriptive accounts of risk and instead clarify the mechanisms through which carceral environments may reproduce distress or, conversely, offer opportunities for protection and support. By foregrounding lived experience within a specific institutional context, the study seeks to offer actionable insights for more inclusive, trauma-informed, and responsive psychiatric care in correctional settings.

## 2 Methods

### 2.1 Study design and approach

This study adopts a case-informed qualitative design to explore the psychiatric, social, and institutional challenges experienced by TGD inmates within the Italian prison system (10). Rather than focusing solely on clinical descriptions, the analysis draws from in-depth case material to identify systemic patterns related to gender identity recognition, psychiatric care, and institutional dynamics. The study is guided by frameworks from social psychiatry and minority stress theory, and incorporates a reflexive, inductive analytic stance to account for the influence of institutional context.

### 2.2 Setting and case selection

All four cases were identified within the special section for transgender inmates in the Belluno prison (Carcere di Belluno)—a correctional facility in Northern Italy known for housing a dedicated area for TGD individuals. This setting allowed for direct and consistent multidisciplinary observation and collaboration between prison staff and external healthcare providers, including the Veneto Regional Reference Center for Gender Incongruence.

Cases were selected based on variation in gender identity trajectories and self-definition, differing engagement with gender-affirming medical and psychiatric care, and relevance to recurring institutional or clinical challenges observed within the Belluno facility. To minimize the risk of coercion inherent to carceral settings, informed consent was obtained by clinicians who were not directly involved in disciplinary decisions or in determining access to gender-affirming treatments. The selection aimed to reflect a diverse range of lived experiences and systemic difficulties within a single institutional context, acknowledging that findings are not meant to be statistically generalizable but rather analytically transferable to similar settings.

### 2.3 Data collection

Data were drawn from multiple sources integrated into routine clinical practice. These included clinical and psychiatric evaluations conducted during incarceration, as well as endocrinological assessments and treatment histories, and relevant administrative documentation (e.g., housing records and clinical correspondence).

Clinical assessments and follow-up interviews were conducted regularly over the course of incarceration, with frequency varying according to individual clinical needs and institutional constraints rather than a fixed schedule. Informal semi-structured interviews took place during routine clinical encounters and follow-up visits.

Data collection was carried out by different members of the multidisciplinary clinical team, including psychiatrists, psychologists, and mental health professionals involved in the care of TGD inmates. All staff conducting interviews had clinical experience in mental health care and received training consistent with gender-affirming and trauma-informed practices through their involvement in specialized regional services and multidisciplinary case discussions.

To enhance transparency, the informal semi-structured interviews were guided by open-ended prompts focusing on key experiential domains, such as: (a) perceptions of housing placement and safety; (b) experiences of gender identity recognition within the institution; (c) access to and continuity of gender-affirming and psychiatric care; and (d) interactions with staff and other inmates. Interviews were flexible and adapted to the clinical context rather than strictly standardized.

Observational data were also collected during multidisciplinary team meetings, which involved professionals from psychiatry, psychology, endocrinology, nursing, and prison healthcare services, further contributing to the contextual understanding of each case.To enhance trustworthiness, data sources were triangulated across clinical documentation, interview material, and institutional observations, allowing emergent themes to be corroborated across contexts.

### 2.4 Analytical strategy

The case material was synthesized using a reflexive thematic analysis approach, with cross-case comparison focused on systemic, institutional, and psychosocial factors. Three key themes emerged through iterative cycles of coding, memo-writing, and team discussion. Coding was conducted independently by multiple members of the research team and discrepancies were discussed collectively to achieve interpretive consensus, thereby strengthening analytic credibility and dependability. An audit trail documenting coding decisions and theme development was maintained throughout the analytic process.

Reflexive consideration of researcher positionality—including clinical experience with gender-affirming care—was integrated to minimize interpretive bias and enhance confirmability. Analytical attention was directed toward patterned mechanisms across cases rather than individual pathology, consistent with case-informed qualitative methodology. Each theme is supported by narrative excerpts and case-based illustrations, emphasizing the institutional determinants of distress, exclusion, and minority stress.

To contextualize the thematic analysis and enhance transparency, we provide a descriptive overview of the four participants included in this study (see **Table 1**). This summary includes demographic characteristics, gender identity trajectories, engagement with gender-affirming care, and psychiatric features relevant to the analysis. All identifying information has been anonymized, and pseudonyms have been used throughout the manuscript to protect participant confidentiality.

**Table 1:** Summary of case characteristics and key themes.

Participant	Age	Assigned sex at birth	Self-identified gender	GAHT use	Diagnosis	Identity stability	Key themes	
M	24	Male	Questioning/fluid	No	Antisocial traits	Low	Identity instability, intra-group tension
S	38	Male	Previously identified as transgender; currently identifies as a gay man	Interrupted/ceased	Cluster B traits	Low	Strategic use of GAHT, housing ambiguity
L	45	Male	Transgender woman	Yes	None reported	High	Affirmation, criticism of group ambiguity
T	25	Male	Transgender woman	Yes	None reported	High	Identity affirmation, housing friction

All names are pseudonyms. Data anonymized.

Thematic saturation was reached when no new institutional mechanisms or categories of distress emerged across cases during iterative coding. Given the sensitivity of prison environments and the restricted size of the TGD population within this facility, the sample reflects the entirety of individuals meeting inclusion criteria during the observation period. In case-informed qualitative research, depth of observation and analytic richness are prioritized over sample size, and the inclusion of four diverse trajectories provided sufficient variation to examine recurrent institutional patterns.

### 2.5 Ethical considerations

Written informed consent was obtained from all participants. The Territorial Ethics Committee “Area Centro-Est Veneto” (CET-ACEV) confirmed that no formal ethical approval was required for this case-informed qualitative analysis, in accordance with Italian regulations for non-interventional observational studies.

## 3 Results

The thematic analysis of the four case narratives from the Belluno prison revealed three central domains that illuminate the systemic and institutional challenges faced by transgender and gender-diverse (TGD) inmates: (1) variability in gender identity recognition and institutional categorization; (2) intra-group tensions within TGD-designated housing sections; and (3) gaps in gender-affirming care and psychiatric support in carceral settings.

To enhance transparency of the analytic process, themes were derived through systematic cross-case comparison, examining how recurring institutional, relational, and clinical patterns manifested across the four trajectories. Each theme was identified on the basis of convergent features observed in more than one case and was refined through iterative discussion within the research team. **Table 2** provides an overview of the key case characteristics underpinning each thematic domain.

**Table 2:** Cross-case features underpinning the three thematic domains.

Case	Gender identity trajectory	Housing experience	Access to GAHT & psychiatric care	Salient institutional issues	Themes contributed to
M	Context-dependent, ambivalent; exploration constrained	TGD section within male facility	Disrupted; limited psychological support	Rigid identity categorization; lack of space for exploration	1, 2, 3
S	Shift in self-definition over time	TGD section within male facility	Initiated then interrupted GAHT	Administrative rigidity; lack of continuity	1, 2, 3
T	Stable transgender identity	TGD section; perceived as legitimate	Delayed but ongoing GAHT; telemedicine	Structural barriers; geographic isolation	1, 2, 3
L	Stable transgender identity; long-standing transition	TGD section; intra-group conflict	Interrupted then resumed GAHT	Ambiguity of placement criteria; care disruption	2, 3

### 3.1 Variability in gender identity recognition and institutional categorization

M initially reported a transgender identification during incarceration and described this self-definition as partly shaped by the institutional context, later expressing uncertainty and ambivalence and reporting limited opportunities to explore and clarify their experience of gender. In M’s account, identity-related narratives appeared context-dependent and difficult to consolidate, and exploration was perceived as discouraged—or met with hostility—by both peers and staff. Rather than providing structured psychological support for identity exploration, institutional processes were experienced as treating gender self-identification as procedurally fixed and administratively consequential. As one participant described, “At first I chose to present myself as transgender, but then I felt forced to keep that position without having the space to understand.” (M).

S similarly initiated gender-affirming hormone therapy (GAHT) during incarceration but subsequently interrupted treatment and reported a shift in self-definition over time, illustrating how identity processes may remain unstable when embedded within rigid administrative frameworks.

By contrast, T (25) and L (45), both transgender women with stable gender identities and a consistent request for gender recognition and care, exhibited more continuous gender trajectories. Despite this stability, the institutional setting still constrained their ability to access appropriate services promptly, particularly due to the remote location of the prison and reliance on telemedicine. As one participant noted, “My identity was never the problem—the problem was that everything took months, and most visits were only online.” (T).

### 3.2 Intra-group tensions and ambiguity within TGD-designated housing sections

Although TGD-designated sections are intended to offer safety and affirmation, the cases revealed significant intra-group tensions rooted in perceived legitimacy, gender expression, and differing pathways of identity development. In particular, conflicts emerged around the presence of individuals whose gender identification was experienced by others as unstable, ambiguous, or no longer aligned with a transgender trajectory.

L and T, both transgender women with long-standing gender-affirming journeys, including consistent use of GAHT and, in some cases, surgical procedures, expressed discomfort with inmates such as M and S. M and S had initially presented themselves as transgender during incarceration but later described shifts in self-definition, without pursuing medical or legal transition pathways. Their continued placement within the TGD-designated section was perceived by some peers as misaligned with the section’s intended function, contributing to tensions and mistrust. As one participant stated, “If someone doesn’t really live as a woman, I don’t understand why they are here with us.” (L).

These accounts point to institutional ambiguity in how gender identity and sexual orientation are operationalized within prison housing policies. M and S, who identified as gay men while having previously explored a transgender identification, were interpreted by peers as accessing the TGD section primarily for protection rather than for reasons of gender affirmation. In the absence of transparent and shared criteria for section placement, these perceptions intensified processes of social exclusion and intra-group conflict. More broadly, the lack of distinct accommodations for sexual minority inmates alongside transgender individuals appeared to blur institutional boundaries between gender identity and sexual orientation.

These dynamics highlight the critical need to differentiate between gender-diverse identities and other queer identities in institutional planning, while ensuring that both groups receive appropriate protection and support without conflating their distinct needs.

### 3.3 Gaps in gender-affirming care and psychiatric support in carceral settings

The third theme centers on the inconsistent provision of psychiatric and medical care, particularly regarding gender-affirming treatment, diagnostic continuity, and individualized psychological support.

Across the four cases, participants experienced varying degrees of disruption in access to GAHT, related to different institutional and clinical factors. These included the absence of an official prescription pathway (as in L’s case), prior use of unregulated hormones before incarceration (S), and logistical barriers linked to the prison’s remote location and limited availability of specialized services (M). These difficulties were compounded by limited staff training in gender-affirming psychiatric care and by the absence of standardized, longitudinal assessment protocols. While some evaluations were conducted via telemedicine, participants described these encounters as insufficient to support continuity of care and therapeutic depth. As one participant reported, “When I arrived here, everything stopped—hormones, visits, everything.” (T).

M and S presented with complex psychological profiles, including difficulties in emotion regulation and interpersonal functioning, yet did not receive sustained or structured psychological interventions tailored to their needs. Their shifting identity narratives appeared to function, at least in part, as adaptive responses to a coercive and highly regulated environment, but these dynamics were not consistently addressed through reflective or integrative psychiatric support. By contrast, L and T experienced greater continuity of care and reported higher psychological stability when access to GAHT and mental health services was maintained, highlighting the protective role of coordinated and affirming clinical pathways.

Taken together, these findings underscore the consequences of failing to integrate gender-affirming care within a broader trauma-informed and socially sensitive psychiatric framework. Reliance on self-declaration in the absence of sustained assessment, follow-up, and multidisciplinary coordination may generate structural inconsistencies, compromising both quality of care and relational dynamics within TGD-designated housing sections.

## 4 Discussion

This case-informed analysis highlights the structural, interpersonal, and psychiatric challenges faced by transgender and gender-diverse (TGD) individuals in an Italian prison facility. The analysis of four incarcerated trajectories reveals key systemic issues, including the rigidity of gender identity recognition processes, tensions within protective units, and the fragmentation of psychiatric and gender-affirming care.

A primary issue concerns the inadequacy of psychiatric and institutional systems in responding to gender identities that are fluid, evolving, or shaped by contextual factors. The experiences of M and S, in particular, illustrate how institutional protocols often rely on binary and fixed models of gender identity, which fail to accommodate the dynamic nature of self-definition in carceral environments. These administrative models are misaligned with the reality that many TGD individuals navigate identity expression in strategic ways to ensure personal safety or access to medical services. Existing literature has documented how correctional institutions tend to impose rigid identity classifications, resulting in significant psychological strain for individuals who do not conform to conventional transition pathways (11, 12). This approach limits the ability of psychiatric services to assess gender-related distress within a developmental and individualized framework and may instead contribute to further stigmatization of ambiguity.

The second theme relates to the internal dynamics within protective housing units. Although these sections are designed to enhance safety and dignity for TGD inmates, they may reproduce exclusionary mechanisms when institutional policies fail to distinguish between gender identity and sexual orientation. In the cases examined, individuals who had pursued medically normative transition pathways expressed distrust or discomfort toward others with more fluid or discontinuous gender trajectories. This pattern aligns with findings from recent research showing that social hierarchies and perceptions of authenticity can emerge within marginalized communities, reinforcing norms that privilege binary, medicalized transition experiences (13, 14). Such dynamics suggest that intra-community tensions are not merely interpersonal, but are shaped and reinforced by institutional environments that reward conformity to dominant gender models (15). The inclusion of gay men alongside transgender women in the same protective unit further contributed to confusion, undermining group cohesion and intensifying perceived illegitimacy among certain members.

The third core issue concerns access to adequate psychiatric and gender-affirming care. All four cases showed evidence of disrupted care pathways, including delays in initiating or resuming hormone therapy, insufficient psychological support, and limited availability of professionals trained in trans-specific mental health. These problems are consistent with findings that correctional systems often lack standardized protocols for gender-affirming treatment, leading to inconsistent access and variable outcomes (11, 16). For inmates with pre-existing vulnerabilities or personality traits associated with emotion regulation difficulties, such as those observed in M and S, these gaps in care may exacerbate distress and limit the capacity for therapeutic engagement. By contrast, the relative emotional stability of T and L, who received more consistent care, underscores the protective role of affirming and developmentally informed interventions. As multiple studies have shown, continuity in gender-affirming treatment is associated with reduced psychological symptoms and better overall mental health outcomes in incarcerated populations (12).

From a broader perspective, this analysis supports the view that incarceration itself can function as a social determinant of mental health, particularly for individuals whose identities are misrecognized or constrained by institutional frameworks. The lack of flexible, context-sensitive psychiatric practices in correctional settings not only limits the ability to respond to diverse needs, but may also reinforce structural stigma and symbolic violence that compound the distress experienced by TGD individuals. These systemic pressures contribute to minority stress, intensify vulnerability, and constrain identity expression within coercive environments.

### 4.1 Implications for policy and practice

The findings of this analysis suggest the need for institutional reform across multiple levels. In particular, housing in carceral settings should be understood not merely as a logistical or security decision, but as a central organizational mechanism shaping safety, identity recognition, access to care, and psychosocial well-being for TGD individuals. Gender-affirming care in carceral settings should be supported by structured diagnostic and support pathways that ensure both medical and psychological continuity. Clear and consistent protocols are essential to prevent interruptions in GAHT and to reduce confusion around eligibility, access, and duration of care.

Housing policies must distinguish between the needs of transgender individuals and those of other queer populations. While both groups may face heightened vulnerability, conflating gender identity with sexual orientation can generate tension and erode trust within protective units. Institutional frameworks must be designed to accommodate fluidity in identity, and to support rather than penalize transitions or detransitions during incarceration.

Equally important is staff training. Personnel working in correctional settings must be equipped with the knowledge and tools to manage gender diversity in a competent, respectful, and trauma-informed manner. This includes recognizing dysphoria, responding appropriately to shifts in identity, and avoiding re-traumatization through misgendering or invalidation. A developmentally attuned approach—embedded in broader institutional practice—may reduce intra-institutional conflict and improve both clinical outcomes and the quality of life for TGD inmates.

At the same time, the findings also call for caution in the implementation of institutional recommendations. Measures intended to enhance protection—such as specialized housing sections or clearer placement criteria—may inadvertently reproduce exclusion or rigidity if applied without sufficient clinical flexibility and individualized assessment. In carceral settings, where identity declarations carry administrative and social consequences, policies that prioritize clarity and categorization may unintentionally constrain exploration or reinforce hierarchies of legitimacy among inmates. A reflexive, context-sensitive approach is therefore essential, ensuring that institutional safeguards remain responsive to evolving identities and do not substitute procedural order for psychological support.

### 4.2 Limitations

This study is limited by its focus on a single institutional setting, the Belluno prison in Northern Italy. While this context allowed for in-depth, multidisciplinary observation, the findings may not be directly transferable to other carceral systems with different administrative structures, resources, or cultural dynamics. Additionally, the data were drawn from clinical records and informal interviews, which—while rich and ecologically valid—are subject to interpretive bias and may not fully capture participants’ internal experiences or longitudinal shifts in identity. Member checking was not conducted, due to ethical and logistical constraints inherent to the carceral setting, including restricted access to participants after data collection and concerns regarding confidentiality and potential coercion.

Moreover, the absence of standardized psychometric tools or structured diagnostic interviews limits the strength of psychiatric conclusions. Nonetheless, the depth of access, consistency of longitudinal observation, and heterogeneity of the selected cases offer meaningful insights into a critically under-researched and vulnerable population.

## 5 Conclusion

TGD inmates represent a uniquely vulnerable population whose psychiatric care is shaped not only by personal history but also by institutional structures, identity politics, and systemic neglect. This case-informed analysis reveals the tension between identity affirmation and institutional classification, the limits of protective housing, and the urgent need for reform in psychiatric and medical care provision. While grounded in a single institutional context, these findings offer transferable insights for carceral systems globally, particularly in navigating the complexities of gender identity recognition and psychiatric care within constrained environments. A truly inclusive, ethical correctional system must account for the complexity of gender identity, recognize intra-group diversity, and adopt affirmative, flexible models of care that prioritize well-being, dignity, and self-determination.

## The Gender Incongruence Interdisciplinary Group (GIIG)

Anna Aprile, Anna Belloni Fortina, Annamaria Cattelan, Alberto Ferlin, Alberto Scala, Angela Favaro, Benedetta Tascini, Bruno Azzena, Camillo Barbisan, Carlo Saccardi, Chiara Ceolin, Claudio Terranova, Corrado Marchese Ragona, Daniela Basso, Elena Campello, Elisa Varotto, Eleonora Vania, Fabrizio Dal Moro, Fabrizio Vianello, Francesco Francini, Francesca Venturini, Giancarlo Ottaviano, Giorgio De Conti, Giovanni Frattin, Giuseppe Sergi, Giulia Musso, Laura Guazzarotti, Lolita Sasset, Marina Bonato, Marina Miscioscia, Marta Ghisi, Massimo Iafrate, Maurizio Iacobone, Michela Gatta, Paolo Meneguzzo, Paolo Simioni, Rossana Schiavo, Rossella Perilli, Sandro Giannini, Tommaso Vezzaro.

## Data Availability

The data analyzed in this study is subject to the following licenses/restrictions: The dataset contains sensitive clinical information related to incarcerated transgender and gender-diverse individuals. To protect participant privacy and prevent potential re-identification, full transcripts and clinical documentation cannot be shared publicly. Access to the dataset can be granted upon reasonable request to the corresponding author, pending appropriate ethical and privacy safeguards. Requests to access these datasets should be directed to Paolo Meneguzzo, paolo.meneguzzo@unipd.it.

## Footnotes

1 https://www.garantenazionaleprivatiliberta.it/gnpl/resources/cms/documents/bbb00eb9f2e4ded380c05b72a2985184.pdf
